# Brain substrates for automatic retrieval of value memory in the primate basal ganglia

**DOI:** 10.1186/s13041-021-00871-x

**Published:** 2021-11-16

**Authors:** Hyoung F. Kim

**Affiliations:** School of Biological Sciences, Seoul National University (SNU), Gwanak-ro, Gwanak-gu, Seoul, 08826 Republic of Korea

**Keywords:** Automatic retrieval, Habit, Value, Long-term memory, Basal ganglia, Decision-making, Automatic behavior, Controlled behavior, Visual object, Macaque monkey

## Abstract

Our behavior is often carried out automatically. Automatic behavior can be guided by past experiences, such as learned values associated with objects. Passive-viewing and free-viewing tasks with no immediate outcomes provide a testable condition in which monkeys and humans automatically retrieve value memories and perform habitual searching. Interestingly, in these tasks, caudal regions of the basal ganglia structures are involved in automatic retrieval of learned object values and habitual gaze. In contrast, rostral regions do not participate in these activities but instead monitor the changes in outcomes. These findings indicate that automatic behaviors based on the value memories are processed selectively by the caudal regions of the primate basal ganglia system. Understanding the distinct roles of the caudal basal ganglia may provide insight into finding selective causes of behavioral disorders in basal ganglia disease.

## Introduction

Animals are constantly moving but are not always intentional or mindful of their movements [1–4]. One part of the body that we can use to study automatic movement is the eyes. The eyes receive visual input from the outside world, and animals, especially primates, rely on visual information for a significant part of their decision-making [5]. The eyes often move automatically to collect visual information.

Notably, the eyes do not simply react to sensory stimuli, but automatic eye movement can be modified based on past experience [6, 7]. For example, automatic eye movement based on experience was observed when the eyes automatically fixated on items related to a simple word such as “tea-making.” [1] The eyes automatically fixated on items related to tea-making, such as a cup, stove, and pot, indicating that memory about the tea-making guides automatic searching. There are other examples of experience-guided automatic eye movements used by experts: the automatic gazes of expert surgeons fixate more on task-relevant areas compared with non-experts or junior surgeons, and professional gamers find valuable objects very quickly and accurately on the screen [8–10]. These examples suggest that repeated training can increase our ability to search for valuable objects and turn the trainee into a visual seeking expert. In the natural world, in which resources are limited, animals compete to find valuable objects faster than others, and these automatic eye movements based on value memory are helpful to maximize reward [11, 12]. It is likely that the expert’s brain automatically retrieves the value map of learned objects to create a state of readiness. Studying how the brain automatically retrieves learned values is a key to understanding how animals successfully guide automatic gazes to valuable objects in achieving a goal.

This review highlights recent advances in the brain mechanisms of primate automatic eye movements based on long-term value memory and provides an idea about the role of caudal region in the primate basal ganglia on automatic value retrieval process.

### Automatic eye movements become a visual habit through object value learning

**Fig. 1:**
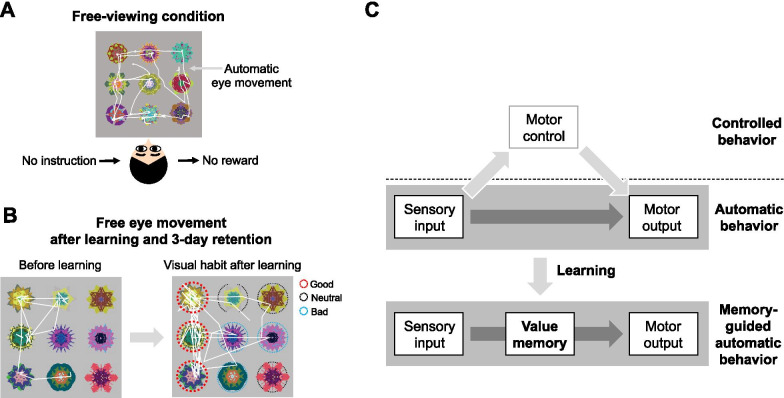
Generation of visual habit after long-term learning. **A** Free-viewing condition. In this condition, there are no instructions and no rewards, allowing human subjects to move their eyes toward presented objects automatically. The white line indicates an eye trace of the human subject. **B** Visual habit: automatic eye movement based on previously learned object values. After long-term learning of object-reward associations and more than 3 days of retention, the eye gazes of human subjects were biased toward previously learned good objects, and the subjects’ gazes occurred without intention or awareness. White lines indicate eye traces of human subjects. Dotted circles indicate the learned values of fractal objects. **C** Memory-guided automatic behavior: habit. Information from the sensory inputs can be sent directly to the motor output structures, generating automatic behavior. Learning changes the brain to remember previous experiences and guide automatic behavior. For example, after long-term learning of object and reward associations, the learned value memory guides automatic behavior for maximizing the reward acquisition

In order to test automatic eye movement to find valuable objects, animals first learned values of visual objects. A group of visual fractal objects was associated with a monetary reward for humans and a liquid reward for monkeys (good objects), whereas other groups of objects were associated with no reward (neutral objects) or the punishment of withdrawing monetary rewards for humans and an air puff for monkeys (bad objects). The object values were learned with an object-value association task in which monkeys and humans chose a visual fractal object associated with liquid and monetary reward, respectively [13]. In the learning task for human subjects, two objects were presented on the screen, and the participants were asked to select one object that was associated with a higher value by making a saccade. After 4 days of learning these object values and a longer than 1-day retention time, the eye movements of human subjects were examined in the free-viewing condition shown in Fig. 1A and B, where no instruction and no rewards were given, so saccades to the objects were meaningless.

Notably, in this free-viewing condition, the gazes of human subjects were biased toward previously learned good objects rather than learned bad and neutral objects compared to gazes before the object value learning (Fig. 1B). The participants reported no active seeking and no control of eye movement in the post-task survey. Monkeys showed the same gaze biases toward previously learned good objects in the free-viewing condition [14, 15]. Humans and monkeys knew that there was no purpose to the objects in the free-viewing condition, while their eyes chose learned good objects automatically. Also, these biased eye movements were maintained for more than a month after learning and were considered a visual habit. These show that the automatic eye movements in free-viewing condition were altered by learning the reward values of visual objects in human and monkey studies [13–15]. We next discuss how the brain automatically retrieves value memory.

### Passive viewing for automatic value memory retrieval

Can memory be retrieved even if the animals do not intend to remember? To produce automatic behavior that contributes to survival, the brain should be able to retrieve automatically an experience learned in the past. This is one of the fundamental concepts to understand how animals behave automatically based on past experience.

**Fig. 2:**
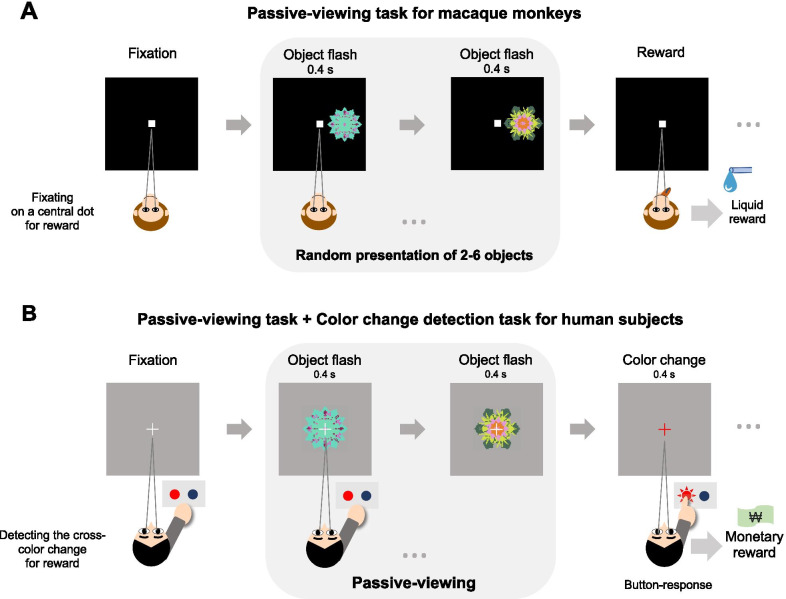
Passive-viewing tasks for investigating automatic memory retrieval in the primate brain. **A** Passive-viewing task for monkeys. After long-term learning of object-reward associations, the learned objects were presented while the monkey fixated on a central white dot. In this task, a reward was not associated with the presented objects. **B** Passive-viewing task for humans. The subject had to report the change in cross color to acquire a monetary reward while previously learned objects were presented. This procedure ensures that the subject focused on the fixation cross and not on the learned objects

Passive-viewing tasks can test whether value memory is retrieved in the brain automatically [15, 16]. The purpose of the passive-viewing task is to provide a task condition to examine neural responses during the incidental perception of learned objects. For monkeys, previously learned objects were sequentially presented while the monkeys fixated on a central white dot (Fig. 2A). No outcome was delivered during the object presentation. Instead, looking at a central white dot was associated with a liquid reward delivered after a random number of object presentations. Thus, the reward was not associated directly with the fractal objects in passive viewing. Because the only reward-acquisition behavior was to gaze at the central white dot, the monkeys did not have to pay attention to the objects that were presented peripherally (Fig. 2A). In this passive-viewing task, in which a reward is provided by fixation on a central dot, it is assumed that value memory for the learned objects is not necessarily retrieved because these objects are meaningless for reward acquisition.

This passive-viewing task for monkeys was modified for human subjects to test further whether memory can be retrieved automatically [13]. The task was designed to test brain responses more strictly than to previously learned values of objects by inserting an unrelated task (color change detection task) during the passive viewing of objects (Fig. 2B). In the passive-viewing task during functional magnetic resonance imaging (fMRI) sessions, the subjects were instructed to pay attention to a center fixation cross to detect a change in its color for a monetary reward, while previously learned objects were presented behind the cross (Fig. 2B). The subjects pressed either the left or right button in response to the cross color changing to red or blue. If the subjects pressed the correct button, a monetary reward was deposited (Fig. 2B). Visual fractal objects learned in the past were shown one by one behind the white fixation cross. In this task, the subjects did not need to be aware of the fractal objects, as they were meaningless for reward acquisition. In a survey after completing the task, the subjects reported that they focused on the change in color of the fixation cross and did not retrieve the objects and their values presented during passive viewing.

**Fig. 3:**
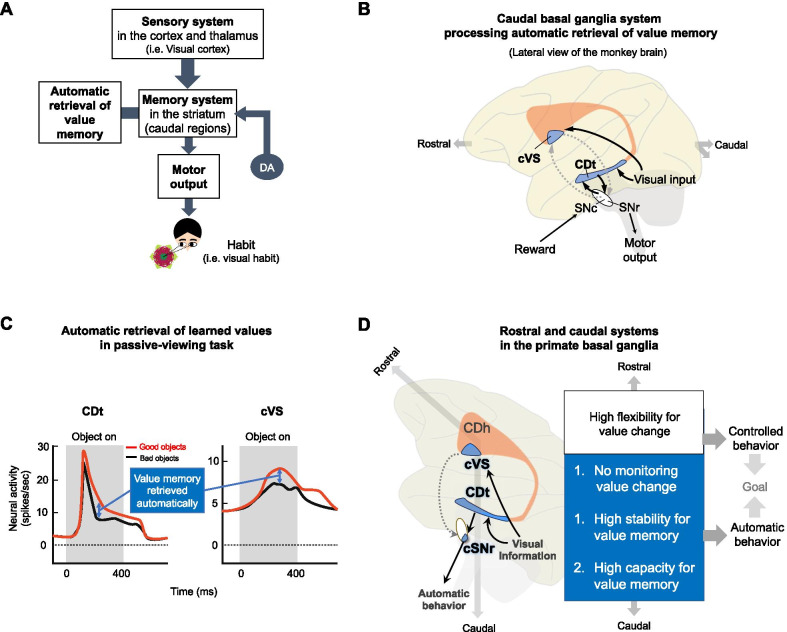
Role of the caudal regions of the basal ganglia in automatic memory retrieval. **A** Location of the striatum between the sensory input and motor output systems, where it plausibly can guide automatic behavior based on value memory. DA: Dopamine neuron. **B** Caudal basal ganglia system processing automatic retrieval of value memory. Previously learned values of visual stimuli were retrieved automatically and selectively in the caudal regions of the primate basal ganglia. Black arrows: anatomical connections confirmed in the primate brain. Gray dotted arrows: anatomical connections to the caudal regions that need to be confirmed in the primate brain. **C** Neuronal representation of automatic memory retrieval in the passive-viewing task. The caudal region of the caudate nucleus (caudate tail, CDt) and caudal region of the ventral striatum (cVS) represent long-term value memory of visual objects retained even several days or several months after the last learning session in the passive-viewing task. **D** Functional properties of the rostral and caudal regions of the basal ganglia. The monkey brain is rotated ~ 45 degrees to position the caudal regions at the bottom. Blue regions indicate the caudal regions of the basal ganglia structures that represent the object value memory retrieved automatically. Black arrows: anatomical connections confirmed in the primate brain. Gray dotted arrows: anatomical connections to the caudal regions that need to be confirmed in the primate brain

To investigate the neural representation of automatic value retrieval, neuronal responses in each brain area to learned objects were examined during passive-viewing task with fMRI and single-unit electrophysiology for humans and macaques, respectively. This passive-viewing task combined with fMRI and single-unit electrophysiology allows investigation of the automatic process of memory retrieval in primate brains. For example, neurons in the caudate tail showed value discrimination activities to learned objects: higher response to learned good objects than to bad objects (Fig. 3C). This difference between neural activities to good and bad objects shows the value memory that is automatically retrieved in passive-viewing task (Fig. 3C). Next, we discuss which regions of the basal ganglia process this automatic retrieval of learned values.

### From where is the value memory automatically retrieved in the brain?

Which regions of the brain process value memory that is automatically retrieved by a stimulus to guide habitual searching? Recent studies have shown that caudal regions of the primate striatum play a role in habitual eye movement (Table 1) [12, 15, 17, 18]. Notably, fMRI and single-unit recording studies with the passive-viewing task showed that caudal regions of the primate striatum represented value memory automatically, but the rostral regions of the striatum did not [15, 19–21].

**Table 1:** Summary of regions showing automatic value retrieval responses in the primate basal ganglia

Region in the basal ganglia	Function	Putative cell type /Target region	Behavioral task	Manipulation		Subject	References
				Method	Effect		
Tail of the caudate nucleus (caudal region)	Automatic value retrieval, Spatial selectivity, Stimulus–response process	Medium spiny neuron / Caudal-ventral SNr, Caudal-ventral GPe	Long-term value learning, Passive-viewing, Free-viewing, Free-looking, Category learning, Motor learning	Inactivation by muscimol injection, Neurotoxic lesion	Deficit in LTVM-guided automatic eye movement, Deficit in visual habit	Macaque monkey, Human	[12, 15–17, 52]
Ventral striatum (caudal region)	Automatic value retrieval	Medium spiny neuron / n/a	Long-term value learning, Passive-viewing, Free-viewing	n/a	n/a	Macaque monkey, Human	[13]
Globus pallidus external segment (caudal-ventral region)	Automatic value retrieval, Spatial selectivity	Medium spiny neuron / Caudal-ventral SNr	Long-term value learning, Passive-viewing, Free-viewing	n/a	n/a	Macaque monkey	[19, 25, 33]
Substantia nigra pars reticulata (caudal-lateral region)	Automatic value retrieval, Spatial selectivity	Medium spiny neuron / Superior colliculus	Long-term value learning, Passive-viewing, Free-viewing	n/a	n/a	Macaque monkey	[21, 34]
Substantia nigra pars compacta (caudal-ventral region)	Automatic value retrieval, Spatial selectivity	Dopamine neuron / Caudate tail	Long-term value learning, Passive-viewing, Free-viewing	n/a	n/a	Macaque monkey	[14, 29]
Putamen (caudal-ventral region)	Automatic value retrieval, Spatial selectivity	Medium spiny neuron / n/a	Long-term value learning, Passive-viewing, Free-viewing, Visual discrimination	Neurotoxic lesion	Deficit in visual habit	Macaque monkey	[12, 53]

Automatic value retrieval neurons were identified by the value discrimination responses in passive-viewing task with macaque monkeys. Value discrimination responses in the human brain regions were examined with functional magnetic resonance imaging. *LTVM* long-term value memory, *GPe* globus pallidus external segment, *SNr* Substantia nigra pars reticulata, *n/a* not available

Are the caudal regions of the striatum plausible regions for processing the value memory for a visual habit? Given the automaticity of habitual gaze that is generated without intentional motor control, sensory information is likely to be sent directly to the motor output system but not to regions of the brain that control and monitor motor movement (Fig. 1C) [7]. The striatum is located between the sensory system and the motor output system (Fig. 3A). Recent studies have shown that caudal regions of the rat and monkey striatum receive direct sensory inputs from the sensory cortex and sensory thalamus and innervate motor output structures in the brain stem [22–26]. For example, visual information of objects from the temporal visual cortex is sent directly to the caudate tail (CDt) located in the caudal region of the monkey striatum (Fig. 3B) [22, 23, 27]. Neurons in the CDt project directly to the substantia nigra pars reticulata (SNr), which is a motor output structure for eye movements (Fig. 3B) [25]. Anatomical connections of the visual cortex-CDt-SNr suggest that if neurons in the CDt process value memory, as shown in Figs. 3A, B, this circuit can guide habitual behavior. Indeed, CDt neurons represented learned value memory of visual objects that were automatically retrieved in the passive-viewing task (Fig. 3C, left) [15, 16]. In addition, recent research has found that the caudal region of the ventral striatum (cVS), which directly receives input from the temporal visual cortex and sends output to the SNr, also processed the value memory that was automatically retrieved in passive-viewing tasks with monkey and human subjects (Fig. 3C, right) [13, 28]. This visual cortex-caudal striatum-SNr connection suggests the direct process of sensory-to-motor response, guided by previously learned values for habitual eye movement (Fig. 3A).

Furthermore, dopamine neurons project strongly to the striatum, including the CDt and VS in monkeys and the tail of the striatum in rats (Fig. 3A, B) [24, 29, 30]. Because dopaminergic projections are involved in the learning process, dopamine neurons might modulate the neurons in the cVS and CDt to encode value memory of visual objects (Fig. 3A) [31, 32]. Notably, a recent study showed that dopamine neurons in the caudal region of the substantia nigra pars compacta (cSNc) selectively represented value memory in the passive-viewing task, similar to cVS and CDt neurons [14]. This suggests that dopamine neurons in the cSNc play a role in value memory maintenance as well as value learning.

### Properties of caudal basal ganglia system for value memory-guided automatic behavior

Anatomical and functional studies have shown that visual information from the cortex is sent to the caudal region of the SNr (cSNr) through the caudal region of the caudate (CDt) through a direct pathway or is sent to the cSNr through the caudal region of the globus pallidus externa (cGPe) and the CDt through an indirect pathway (Fig. 3B) [19, 25, 33]. Neurons in these caudal areas of striatal structures represented previously learned values of visual objects in passive-viewing tasks [15, 19, 34]. Long-term value memory processed in cSNr neurons controls neural activity in the superior colliculus (SC) to guide habitual eye movement. Impairment in visual habit by CDt inactivation provides evidence for a critical role of the caudal basal ganglia circuits in automatic eye movement based on past experience (Table 1) [15]. In addition, neurons in the cVS, cSNc and other basal ganglia structures in the caudal region were found to represent long-term value memory in the passive-viewing task (Table 1) (Fig. 3C) [34]. However, the rostral regions of the basal ganglia structures did not represent long-term value memory for habitual behavior [15, 21]. Taken together, these data suggest selective involvement of caudal areas of the basal ganglia system in value memory-guided automatic eye movement.

What properties of this automatic eye movement can be explained by the caudal regions of the basal ganglia structures? Visual information from the visual areas is sent to the CDt and cVS, and then it projects directly to the motor output structure, the cSNr (Fig. 3D). This direct sensory-to-motor circuit of the visual cortex-CDt/cVS-cSNr is likely to process direct sensory stimuli-inducible motor responses without monitoring of value change (no monitoring the value change) (Fig. 3D). Second, memory-guided automatic behaviors, such as habits, are known to be persistent [35, 36]. Because these behaviors are guided by memories of previous experiences, these memories have to be retained in the brain. Indeed, memories represented in caudal regions of the basal ganglia structures are maintained stably for more than several days or even for more than a year after learning in monkey and human studies [14, 15, 34, 37]. Thus, the caudal basal ganglia circuits are likely to guide habitual behaviors with these stably maintained memories (high stability for value memory) (Fig. 3D).

In contrast to the properties of the caudal basal ganglia structures, rostral regions of the basal ganglia are involved in flexible value memory that processes changes in reward value [15, 21, 38, 39]. In reversal tasks, where one of two objects in a block was associated with reward and the other was not and this association was reversed in a following block, neurons in the rostral region of the caudate nucleus [caudate head (CDh) indicated in Fig. 3D] showed a higher response to the reward-associated object in each block [15]. This value memory flexibility to track the change in the reward outcome is suitable to control behavior in accordance with the environmental changes (high flexibility for value change and controlled behavior) (Fig. 3D).

Finally, neurons in caudal regions of the basal ganglia structures represent previously learned values of hundreds of objects (high capacity for value memory) [34, 40]. Because the value memory in the caudal regions of the basal ganglia is not as versatile as that in the rostral regions, new information is likely to accumulate in previous memory storage, suggesting that the caudal regions should have high capacity for value memory, allowing guidance of a large part of an animal’s automatic behavior (Fig. 3D).

### Implication in basal ganglia disorders

Previous monkey studies showed that caudate tail inactivation by muscimol injection impaired automatic gaze bias to learned good objects, suggesting that impairment of the caudate tail may lead to behavioral disorders in patients with basal ganglia diseases (Table 1) [15]. Interestingly, it is reported that neuronal degeneration initially occurs in the caudate tail in Huntington’s patients [41, 42]. These patients have difficulty in finding a target object in a visual search task [43–45]. These studies suggest that impairment in the caudate tail may cause the malfunction of automatic retrieval process, which may eventually lead to a disorder in the automatic searching in Huntington’s patients.

In addition, abnormal levels of dopamine in the basal ganglia are known to be a critical factor in causing behavioral disorders [40]. Dopamine deficiency in the striatum is well known to cause Parkinson’s disease, which is associated with motor symptoms such as tremor, bradykinesia, and rigidity, as well as cognitive symptoms such as difficulty in learning of probabilistic classification [46, 47]. In contrast, high levels of dopamine in the striatum are thought to be related to drug abuse, addiction, and hallucination [48, 49]. Interestingly, recent study showed that hallucination-like perception can be induced by optogenetic boosting of dopamine selectively in the tail of striatum (TS) in caudal basal ganglia system of mice [24, 50]. It is plausible that this increase in dopamine level may boost the automatic retrieval process of previously experienced sensory information in TS, generating non-selective actions without regard for context [51]. So far, there are a few studies about the involvement of the caudal basal ganglia system in brain disorders. In order to understand region-selective disorders in basal ganglia diseases and to find the disorder-selective treatment, mechanisms on how caudal and rostral regions of the basal ganglia differentially generate motor, cognitive, and psychotic disorders will be investigated with the passive-viewing and free-viewing paradigms in the future.

## Conclusion

Recent studies with the human and monkey passive-viewing tasks show that learned values of objects are automatically retrieved in caudal regions of the basal ganglia system. The rostral and caudal regions of the basal ganglia allow animals to adapt to changing environments with monitoring the changes in value and to achieve a goal quickly and accurately with automatic behavior based on sustained value memory in a normally stable environment, respectively (Fig. 3D). This selective participation of caudal basal ganglia in automatic memory retrieval provides insight to investigate the brain mechanisms of automatic behavior such as habit and link them to unconscious behavior and basal ganglia disorders.

## Data Availability

Not applicable.

## Abbreviations

LTVM: Long-term value memory
CDh: Caudate head
CDt: Caudate tail
SNr: Substantia nigra pars reticulata
SNc: Substantia nigra pars compacta
VS: Ventral striatum
GPe: Globus pallidus external segment
TS: Tail of striatum
